# Recent Advances in Graphene Oxide Membranes for Gas Separation Applications

**DOI:** 10.3390/ijms20225609

**Published:** 2019-11-09

**Authors:** Saif Khan Alen, SungWoo Nam, Seyed A. Dastgheib

**Affiliations:** 1 Department of Mechanical Science and Engineering, University of Illinois at Urbana-Champaign, 1206 West Green Street, Urbana, IL 61801, USA; salen2@illinois.edu (S.K.A.); swnam@illinois.edu (S.N.); 2 Illinois State Geological Survey, Prairie Research Institute, University of Illinois at Urbana-Champaign, 615 East Peabody Drive, Champaign, IL 61820, USA

**Keywords:** membrane, gas separation, graphene oxide, hydrocarbon separation, gas purification

## Abstract

Graphene oxide (GO) can dramatically enhance the gas separation performance of membrane technologies beyond the limits of conventional membrane materials in terms of both permeability and selectivity. Graphene oxide membranes can allow extremely high fluxes because of their ultimate thinness and unique layered structure. In addition, their high selectivity is due to the molecular sieving or diffusion effect resulting from their narrow pore size distribution or their unique surface chemistry. In the first part of this review, we briefly discuss different mechanisms of gas transport through membranes, with an emphasis on the proposed mechanisms for gas separation by GO membranes. In the second part, we review the methods for GO membrane preparation and characterization. In the third part, we provide a critical review of the literature on the application of different types of GO membranes for CO_2_, H_2_, and hydrocarbon separation. Finally, we provide recommendations for the development of high-performance GO membranes for gas separation applications.

## 1. Introduction

Among the available energy-intensive gas separation technologies, membrane technologies have attracted great interest because of their potential to improve separation performance, lower energy and capital costs, reduce the equipment size, minimize the environmental footprint, and provide easier operation. At present, high-performance gas separation membranes are critical to several industrial applications, such as oxygen production, post-combustion carbon capture, natural gas purification, and synthesis gas (syngas) processing. The performance of gas separation membranes, which can be characterized in terms of permeability and selectivity, is primarily dependent on the physical and chemical properties of the membrane materials [1,2,3,4,5,6,7]. Permeability is the ability of the membrane to allow transport of gas molecules, whereas selectivity is the ability of the membrane to identify and selectively allow transport of the desired molecules. The permeability and selectivity of the membranes depend on the physical and chemical characteristics of the membrane materials. Membrane materials can be categorized into three main groups: dense, porous, and asymmetric membranes [8]. The mechanism of gas transport through these membranes depends on the type of material (e.g., whether the material is dense or porous). As discussed later in Section 2, there are four main transport mechanisms: solution diffusion, Knudsen diffusion, capillary condensation, and molecular sieving.

During the last few decades, polymer-based membrane technologies have developed sufficiently to provide selective gas separation performance with a reasonable amount of permeability. The bounds of performance are governed by the Robeson limit [9], which states that the permeability of polymeric membranes must be sacrificed to obtain higher selectivity and vice versa, which limits the performance of these membranes in gas separation applications. Polymeric membranes are presently used extensively, although nanoporous inorganic membranes have also been developed with different suitable materials, such as silica, zeolite, metal–organic frameworks (MOF), and perovskite. The performance of both polymeric and nanoporous membranes is limited by several factors, including the minimum thickness of the membrane (to maximize the flux), the maximum achievable selectivity, and their robustness at higher temperatures and pressures or under corrosive and reactive conditions. Along with researchers’ continuing development of the performance of existing membrane materials, recent progress has been aimed at developing new membrane materials that will enhance their gas separation performance beyond the present limits. 

Graphene and its derivatives are promising materials for the development of a new generation of membranes, mainly because of their ultimate thinness, scalable two-dimensional (2-D) nature, and excellent membrane formation capabilities [7,10,11,12,13,14,15,16]. Graphene oxide (GO), an atomically thin monolayer of graphite oxide, is a wrinkled [17] 2-D carbon sheet that contains various oxygenated functional groups on its basal planes and at the edges. The thickness of monolayer GO is approximately 1 nm, whereas its lateral dimension may vary from a few nanometers to several micrometers. Unlike graphene, GO is functionalized with bulky oxygen-containing groups, which results in greater interlayer spacing (~0.6–1.0 nm) of a stacked GO film. Because of the thinness and narrow interlayer spacing of layered GO membranes, they can provide very high selectivity as well as rapid permeation in gas separation applications. 

Graphene without any structural defects does not allow the transport of even small gas molecules through its surface [18]. Therefore, graphene materials must be made porous for use in membrane separation, which can be accomplished by two approaches. In the first approach, nanoscale pores can be created in the graphene surface (e.g., by laser/ion beam [19] drilling or plasma oxidation [20]) to obtain a molecular sieving effect. In the second, ultrathin flakes of graphene materials can be stacked such that gas can be transported through the interlayer spacing. A combination of these two approaches is also possible by having the gas transported vertically through defect holes on the graphene sheets and horizontally through the nanochannels formed in the stacked graphene layers. Fabricating nanopores with precise diameters over a graphene layer is a difficult task. Randomly stacked graphene films also have very small average interlayer spacing (~0.355 nm), which is not favorable for gas molecules to pass through [2]. In contrast, the interlayer spacing of a stacked GO film (~0.6–1.2 nm) is much larger than that of graphene depending on the humidity, which makes it a better candidate for gas separation applications.

Recent review articles (e.g., [7,21,22,23]) discuss the preparation and application of two-dimensional materials, including GO-based membranes, for various separation applications. However, in the present article we present a more focused perspective on gas separation applications of GO-based membranes. First, we briefly discuss different mechanisms of gas transport through membranes, with an emphasis on the proposed mechanisms for gas separation by GO membranes. Second, we review the methods reported for preparing and characterizing GO membranes, including the methods of preparing supported, self-standing, and nanocomposite membrane materials. Third, we focus on a critical review of literature reporting the application of different types of GO membranes for CO_2_, H_2_, and hydrocarbon separation. Finally, we summarize the main challenges and opportunities of emerging GO membrane technologies and provide recommendations for developing high-performance GO membranes for gas separation applications. 

## 2. Mechanisms of Gas Transport through Membranes

**Figure 1:**
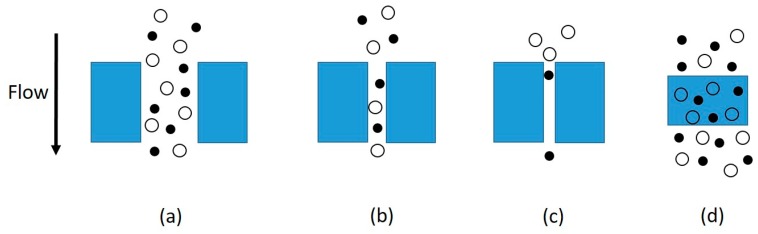
Diffusion mechanisms: (**a**) bulk flow through pores; (**b**) Knudsen diffusion through pores; (**c**) molecular sieving; (**d**) solution diffusion through dense membranes [24]. (Reprinted with permission of Wikimedia Commons under Creative Commons license CC BY-SA 4.0.).

Conventional membranes can be classified into three major groups: dense, porous, and asymmetric membranes. Gas transport through a dense membrane (i.e., without pores) can be described by the solution–diffusion mechanism. This mechanism involves three primary steps: adsorption at the upstream (feed) boundary, diffusion through the membrane, and desorption on the downstream (permeate) side. The gas transport mechanisms through a porous membrane include molecular sieving, Knudsen diffusion, capillary condensation, and laminar flow (e.g., Poiseuille flow), depending on the membrane pore size and diameter of gas molecules. Different mechanisms of gas transport through membranes are shown schematically in Figure 1 [24]. The contribution of each of these mechanisms varies depending on the physicochemical properties of the membrane, the properties of the gases, the molecular interactions between the gas molecules and membrane surface functionalities, and the operating conditions. For example, in the Knudsen diffusion mechanism, where the pores are larger than the size of gas molecules but smaller than the gas mean free path, the gas permeation rate varies inversely with the square root of the molecular weight of the gas, whereas in molecular sieving, only the molecules having a diameter smaller than the pore size of the membrane can permeate, which results in a very high separation factor between the smaller and larger gas molecules [8]. When pores are larger than the gas mean free path, the gas transport is governed by laminar flow and that is proportional to the reverse of the gas viscosity. More details on the fundamentals of transport through membrane pores are provided in a recent review article [16]. Asymmetric membranes are combinations of dense and porous membranes; they consist of a thin, dense barrier layer to provide selectivity and a thick, porous substrate layer to provide physical support to the membrane [8].

**Figure 2:**
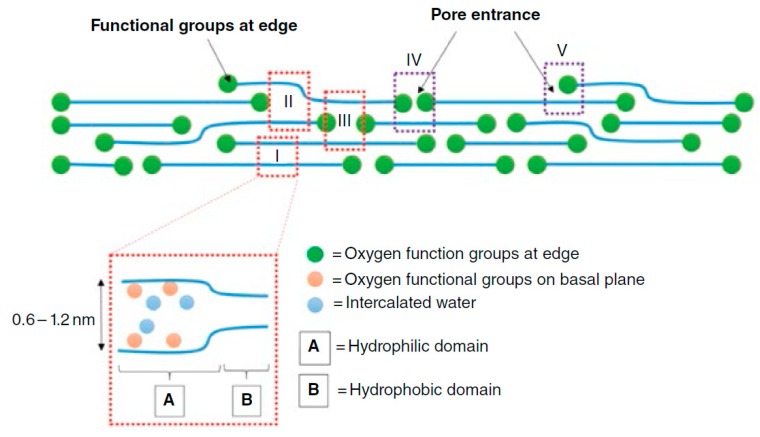
Simplified schematic diagram of a layered graphene oxide membrane structure with functionalized pore edges [7]. Various types of pore entrances are shown by the dotted boxes, identified by roman numerals I–V. (From Park, H.B.; Yoon, H.W.; Cho, Y.H. Graphene oxide membrane for molecular separation. In *Graphene Oxide: Fundamentals and Applications*; Dimiev, A.M., Eigler, S., eds.; John Wiley & Sons: New York, 2017; pp. 296–313. © 2017 John Wiley & Sons, Ltd. Reprinted with permission.)

It has been proposed that gas molecules are transported through GO membranes through nanochannels between the layered GO sheets or through holes created by defects on the GO surface. Nair et al. [1] reported that layered GO membranes can provide unimpeded permeation of water but can block the transport of small gas molecules, even helium. This impeded gas barrier property of the GO membrane in a dry state is due to the reversible narrowing of capillaries in a GO membrane at low humidity, which blocks the diffusion of molecules through the membrane pore entrances (see Figure 2 for an illustration of a layered GO membrane with functionalized pore entrances). The permeation rate of water is significantly faster (10^10^ times) than that of He because of the low-frictional flow of a monolayer of water through the 2-D capillaries formed by closely packed 2-D sheets [1]. The edges of GO are hydrophilic in nature, whereas the planes are hydrophobic, which provides a very rapid permeation of water through multilayered GO membranes. Several oxygen functional groups (e.g., hydroxyl, carboxyl) that are attached to the graphene sheets are responsible for the relatively large interlayer spacing in the GO film. These groups tend to cluster, leaving a large, percolating region of graphene sheet unoxidized. Therefore, the GO laminates are likely to have empty spaces formed between oxidized regions of graphene sheets, forming a network of pristine graphene capillaries within the GO film. Li et al. [3] speculated that selective structural defects in the GO flakes, rather than the interlayer spacing of the GO flakes, provide the major transport pathways for small gas molecules in a GO film. Kim et al. [2] reported that GO membranes are permeable to small gases, even in a dry state, when sufficient transmembrane pressure is applied to overcome the energy barrier to pore entry and diffusion within the pores. The overall contribution of structural defects or interlayer spacing in providing transport routes for gas molecules depends on the physicochemical properties (e.g., size, chemistry) of gas molecules and the characteristics (e.g., size, orientation, and distribution of surface functionalities) of the stacked GO flakes and may vary for different gases and GO membranes (prepared under different conditions). Water molecules clustered on oxygen functional groups at pore entrances may act as integral structural components of the GO membrane, connecting the GO flakes to bring compactness to the membrane, which blocks the transport of gas molecules through the membrane. Upon partial removal of the moisture content by controlled heating or vacuum drying, partially porous membranes can be obtained. Therefore, the water content of a GO membrane or the moisture content of a gas mixture can play major roles in the selectivity and permeability of the GO membrane for the separation of different gas components found in a gas mixture. The water vapor content of a gas mixture may need to be controlled (e.g., by condensation or a drying pretreatment) to optimize the performance of GO membranes for practical applications.

One of the most important characteristics of the layered GO membrane is the interlayer spacing between GO sheets, which can be tailored for optimal transport of the desired molecules through the membrane. The interlayer spacing of the GO membrane can be reduced by the controlled reduction of GO to partially remove the bulky oxygen functionalities. The interlayer space of the GO membrane can also be increased by the intercalation of different ions [25,26,27,28]. Nair et al. [1] performed GO membrane annealing at 250 °C in a hydrogen–argon atmosphere to reduce the interlayer spacing from 1.0 to 0.4 nm, which had a remarkable effect on the gas transport behavior of the membrane. Similarly, Kim et al. [2] obtained a permeable and highly selective GO membrane after performing a simple heat treatment at 140 °C. The thermal annealing caused the formation of irreversible pores, possibly attributable to the removal of water molecules, which made the GO active layers more porous. Li et al. [3] performed a reduction of their GO membrane to effectively narrow the interlayer spacing and limit the permeation of specific molecules. More recently, Qi et al. [5] developed a partially reduced GO membrane that could produce a molecular sieving effect because of the reduced interlayer spacing between the GO flakes. Thermal annealing under an inert or reductive atmosphere is a simple approach for removing surface oxygen functionalities; however, it may damage the structure of GO layers and create large cracks, leading to membrane failure. Thermal treatment can lead to the dissociation of oxygen functional groups, releasing CO_2_ or CO gases that may create additional defects and pores and affecting membrane selectivity and permeability. The quick release of water vapor and gases during thermal annealing may result in the rupture of GO films.

## 3. Preparation and Characterization of GO Membranes

In this section, after briefly reviewing methods of GO preparation, we describe various methods used for the fabrication and characterization of GO membranes. Graphite oxide was first prepared by Brodie in 1859 [29] by oxidizing powdered graphite. Then in 1898, Staudenmaier improved Brodie’s method by adding KClO_3_ in multiple aliquots during the oxidation of graphite in a fuming HNO_3_ [10]. Later, Hummers proposed a modified method for oxidizing graphite by treating it with a water-free mixture of concentrated sulfuric acid, sodium nitrate, and potassium permanganate [30]. In 2010, Marcano et al. [31] proposed improving the method of Hummers by preparing GO in a safer way, by using H_2_SO_4_/H_3_PO_4_ in a 9:1 volume ratio as a mixed acid with the strong oxidant KMnO_4_. Graphene oxide can be obtained by exfoliating graphite oxide through an ultrasonic treatment of GO powder in water, followed by the separation of fine colloidal GO sheets by centrifuge, filtration, or both. Note that GO can also be prepared by oxidizing graphene prepared by the chemical vapor deposition method, although this is a more difficult and expensive option. Various methods of preparing GO membranes from GO are reviewed in Section 3.1, and methods of characterizing these membranes are described in Section 3.2.

### 3.1. Preparation of GO Membranes

Graphene oxide membranes are comparatively simple to prepare from the aqueous dispersion of GO flakes. Several methods are used to prepare GO membranes, such as the filtration-assisted method, evaporation-assisted method, casting- or coating-assisted method, layer-by-layer (LBL) assembly method, templating method, and shear-induced alignment method. In the filtration-assisted method, GO membranes can be obtained by either vacuum- or pressure-assisted filtration. The filtration-assisted method is widely used because of its ability to produce large-scale self-standing GO membranes as well as easy control over the thickness and microstructure of the membranes by adjusting the concentration and volume of the GO solution. Dikin et al. [32] prepared large-scale self-standing GO membranes by using a flow-directed assembly method, and they showed that GO nanosheets are arranged together in a near-parallel way. In the casting- or coating-assisted method, GO membranes can be prepared by several approaches, such as drop casting, drop coating, dip coating (also known as the Langmuir–Blodgett [LB] approach), and spin or spray coating. In the drop-casting approach, a GO colloidal suspension is drop cast onto a substrate with a smooth surface, such as silica or paper, and dried at room temperature, whereas in spin or spray coating, a GO colloidal suspension is spin or spray coated on a smooth substrate, such as a Cu foil or a polymeric support, to fabricate a thin and uniform GO membrane. 

In the review of methods for the preparation of GO membranes, we provide examples from the literature for the preparation of GO membranes for gas separation applications. Kim et al. [2] reported preparing a GO membrane over a polyethersulfone (PES) support by using the spin-coating method. The prepared membrane had superior gas separation performance compared with the existing upper bound of the gas separation membranes [9]. In the LB approach, the GO suspension is first slowly spread onto the surface of the water at a certain speed up to a certain volume, at which point a film with a faint brown-colored GO can be observed at the end of the compression. The GO monolayer is then transferred by vertically dipping the substrate into the trough and slowly pulling it up. Among various methods for preparing GO membranes, the LBL assembly method has attracted substantial research interest because of its promise of providing a compact structure in solutions and good separation performance in water purification applications. In the LBL assembly method, electrostatic attraction and hydrogen bonding make it possible to alternately deposit polymer and GO flakes over a substrate. Graphene oxide membranes that are developed by LBL assembly can enhance the CO_2_ separation process because of the strong CO_2_ affinity of numerous polar groups and the facilitation of CO_2_ transport by amine groups in polyelectrolyte layers [33]. Heo et al. [33] proposed an LBL self-assembly method for enhanced CO_2_ gas separation performance, which is a multilayer deposition process that provides precise control of film thickness and internal structure through complementary interactions, such as electrostatic interactions, covalent interactions, and hydrogen bonding. Table 1 presents a summary of different methods for preparing GO membranes.

For gas separation applications, in addition to the above-mentioned conventional GO membrane preparation methods, researchers have proposed minor modifications of the main conventional methods to obtain the desired thinness and enhanced gas separation performance. Kim et al. [2] prepared PES-supported ultrathin (3–10 nm) GO membranes by using two different approaches of the spin-coating method. In their first approach, before the spin-coating stage, they contacted the support membrane surface with the air–liquid interface of the GO solution, which created a multi-layer GO membrane over the PES membrane. In another approach, they adopted direct-drop spin-casting of the GO solution on top of the PES membrane, which provided highly interlocked GO thin layers. They were able to obtain a highly interlocked thin layer because the carboxylic groups form a negative charge at the edges of the GO sheets, which creates an island-like assembly of GO sheets resulting from the repulsive interaction at the edges. During spin coating, this repulsive edge-to-edge interaction is overcome by the attractive face-to-face capillary force, which results in highly interlocked GO layers. Similarly, Li et al. [3] modified the filtration-assisted method by using two different approaches to prepare ultrathin (~1.8 nm) GO membranes over an anodic aluminum oxide (AAO) support. In one approach, they prepared GO membranes by the vacuum filtration method and found that centrifugation and dilution of the GO dispersions were important parameters to control to obtain high-quality GO membranes. In the second approach, they prepared GO thin film composite membranes by dropping a constant volume of GO solution directly onto a spinning polymer support, which resulted in a highly interlocked brick model with a relatively higher number of intercalated water molecules between the GO layers. 

Researchers have applied other approaches for preparing GO membranes by targeting the specific physical or chemical properties of the membrane. Bouša et al. [4] used a gravitational assembly method to prepare self-standing thin GO membranes. Moreover, Chi et al. [34] reported a facile three-step approach toward GO membrane fabrication: (1) mild freeze–thaw exfoliation of GO to obtain large GO nanosheets with a lateral dimension of 15 µm and a thickness of 2 nm, (2) purification through pH adjustment (e.g., pH 3.0 using 1 M HCl), and (3) spin coating to fabricate smooth GO membranes with uniformly aligned GO nanosheets. In addition to controlling the flake size and thickness of the GO membrane by the above-mentioned approaches, controlling the interlayer spacing of the GO nanosheets is important, as it can be useful for controlling the precise molecular sieving effect of the membrane. Graphene oxide membranes can be reduced to partially remove the bulky oxygen functionalities, which are mainly located at the edges of the GO nanosheets, and reduce the interlayer space. Partial reduction of GO can reduce the interlayer spacing of the GO layers to less than the interlayer spacing of dry GO flakes, which is 0.43 nm [5]. This approach can be used to tailor GO membranes for different molecular-sieving gas separation applications. Graphene oxide can be reduced by reducing the GO powder that is used for GO solution preparation, reducing the GO membrane during fabrication, or reducing the GO membrane after fabrication. Each reduction approach has its specific challenges; for example, reducing the GO powder can significantly change the properties of GO (e.g., the solubility of colloidal GO particles) and cause difficulty in fabricating the GO films, or a postfabrication reduction can cause breakage of the membrane. To avoid these pre- or postfabrication reduction issues, Qi et al. [5] adopted an in situ partial reduction technique during the formation of the membrane. They prepared ultrathin, defect-free GO layers on porous stainless steel hollow fibers (PSSHF) by an electrophoresis deposition (ED) method, in which the electrolytic setup consisted of an electrochemical station, Teflon-supported stainless steel tube (counter electrode), and PSSHF (working electrode). 

**Figure 3:**
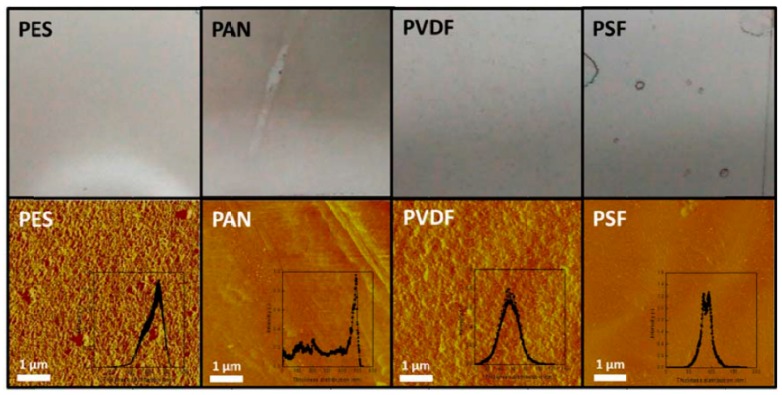
Images of the GO-coated polymeric membranes (top row) and the surface morphologies and thickness distributions (bottom row) of various polymer membranes [2]. PES, polyethersulfone; PAN, polyacrylonitrile; PVDF, poly(vinylidene fluoride); PSF, polysulfone. (From Kim, H.W.; Yoon, H.W.; Yoon, S.-M.; Yoo, B.M.; Ahn, B.K.; Cho, Y.H.; Shin, H.J.; Yang, H.; Paik, U.; Kwon, S.; Choi, J.-Y.; Park, H.B. Selective gas transport through few-layered graphene and graphene oxide membranes (Supplementary Materials). *Science* 2013, *342* (6154), 1–20. Reprinted with permission from AAAS.).

Proper selection of the supporting material is critically important in the development of suitable GO-supported membranes. To illustrate, Kim et al. [2] presented images of GO-coated polymer membranes and showed their surface morphologies along with their thickness distributions (Figure 3). A homogeneous GO coating on the membrane surface is favorable when the surface is less hydrophobic and comparatively smooth. We can observe from the images in Figure 3 that the PES and polysulfone (PSF) polymers provide less surface roughness compared with other support materials tested. Of these two membranes, PES (with a water contact angle [WCA] of 45° ± 2°) is comparatively less hydrophobic than PSF (with a WCA of 75° ± 5°), which creates better conditions for membrane wetting and the deposition of hydrophilic GO nanosheets on the support material. Therefore, the PES-supported GO membrane appears to have no visible nonuniformity and defects. Moreover, PES can withstand a much higher temperature (130 °C), which makes it suitable for any high-temperature membrane application. 

In addition to the development of supported and self-standing GO membranes for gas separation, mixed-matrix membranes (MMM) with GO as a nanofiller have attracted substantial interest among researchers. Mixed-matrix membranes can be prepared by using several approaches, such as LBL assembly [33,35], aqueous GO dispersion [36], or interfacial polymerization [37]. Yang et al. [35] prepared alternately deposited polyethylenimine (PEI)-GO by using an LBL method to investigate the O_2_ barrier of thin film assemblies. Along with the advancement of GO-based MMM, the intercalation of different MOF between GO sheets is widely used for enhancing the gas separation performance of the membranes, which results from the enhanced gap between GO layers. Jia et al. [38] prepared a GO membrane with an intercalated MOF (UiO-66-NH_2_) by vacuum filtration of a suspension solution of UiO-66-NH_2_ and GO on a mixed cellulose ester filter. This modification resulted in enhancing the H_2_ separation performance, which can be explained by the close interfacial contact between the MOF and GO arising from hydrogen bonding and the electrostatic force, resulting in the creation of proper pores for hydrogen separation. Some additional methods, such as ultrasonic exfoliation and vacuum suction, are available in the literature [39,40].

### 3.2. Characterization of GO Membranes

It is vital to characterize both the structural (size, shape, stacking manner, and defects in GO nanosheets) and the chemical (C/O ratio, bond density, and chemical composition) characteristics of the GO membranes. Scanning electron microscopy (SEM), transmission electron microscopy (TEM), and atomic force microscopy (AFM) can provide information related to the uniformity, surface morphology, and surface roughness of the membrane, whereas X-ray diffraction (XRD), X-ray photoelectron spectroscopy (XPS), Raman spectroscopy, Fourier-transform infrared spectroscopy, WCA, surface zeta potential (surface charge), and thermogravimetric analysis (TGA) can provide information about the chemical composition, surface functionalities, surface chemistry, and thermal stability. Table 2 summarizes different characterization techniques adopted by various researchers to characterize the morphology and chemical composition of GO membranes. Table 2 also summarizes the major findings of each work, which include GO flake dimensions, characteristics of carbon bonds, and surface functionalities, among others.

The surface morphology of a GO membrane (i.e., surface texture and size or shape of the GO flakes) can be characterized by SEM. Transmission electron microscopy can be used to evaluate the effectiveness of the exfoliation process by measuring the surface morphologies of the GO flakes. Atomic force microscopy images provide a height profile diagram of the membrane surface, which is important for measuring the depth of the membrane surface and characterizing the surface roughness, as well as for measuring the thickness of the GO layers. 

For GO membrane characterization, Raman spectroscopy is used to identify the chemical composition of the GO or to confirm the existence of a GO layer over the support materials. The content of *sp*^2^- and *sp*^3^-hybridized carbon bonds can be determined by Raman spectroscopy. Two major bands and additional minor bands can be observed in the Raman spectrum of a typical GO membrane: a D band at 1350 cm^−1^ (associated with defects in the regular network of *sp*^2^-hybridized carbon atoms), a G band at 1585 cm^−1^ (associated with the vibration of *sp*^2^-hybridized carbon atoms), a relatively weak 2D band (at 2690 cm^−1^), and other minor bands. 

The intensity of XRD can specify the crystalline structure of the GO nanosheets, indicate the successful synthesis of GO, and be used to calculate the interlayer spacing between GO nanosheets (*d* spacing). As an example, Bouša et al. [4] used XRD to measure the *d* spacing in GO nanosheets. They found one peak at ~10° and estimated the *d* spacing as 8.93 Å. 

X-ray photoelectron spectroscopy can provide the surface chemical composition and characteristics of the oxygen surface functionalities. As an example, Li et al. [3] and Qi et al. [5] used XPS to detect surface elements or the C/O ratio in characterizing their GO membranes. Because of the availability of an extensive database for binding energies of various carbon–oxygen functionalities, it is also possible to characterize the distribution of various oxygen functionalities on GO membranes through deconvolution of the XPS spectra. 

Fourier-transform infrared spectroscopic analysis can be used to identify various oxygen functional groups based on the stretching vibrations of different bonds. These have included O–H (3400 cm^−1^), C=O (1720 cm^−1^), C=C skeletal (1620 cm^−1^), C–O carboxyl (1390 cm^−1^), C–O epoxy (1195 cm^−1^), and C–O alkoxy (1035 cm^−1^), which were detected on the surfaces of GO membranes [4]. 

Several other important tools can be used for GO membrane characterization, such as tensile strength measurement, combustible elemental analysis, dynamic light scattering (DLS), TGA, and WCA. Chi et al. [34] used DLS to analyze the particle size distribution of a GO solution. Wu et al. [41] used TGA to characterize a GO and a GO composite membrane at a heating rate of 10 °C min^−1^ under a N_2_ atmosphere. A weight variation with a temperature increase indicates the release of free water (below ~100 °C), the release of entrapped water in micro- and nanochannels (between 100 and ~200 °C), and the decomposition of various surface functionalities or the substrate at higher temperatures. Qi et al. [5] observed that the reduction of a GO membrane could reduce the interlayer spacing. Zheng et al. [42] developed a membrane characterization technique that used an integrated quartz crystal microbalance with dissipation and ellipsometry to quantify the interlayer spacing of a GO membrane in an aqueous environment. In this approach, to measure the *d* spacing and swelling of a GO membrane in aqueous solution, they integrated the two methods (quartz crystal microbalance with dissipation and ellipsometry), which allowed them to simultaneously monitor changes in the mass and thickness of a layered GO film. 

## 4. Gas Separation Performance of GO Membranes

The gas separation performance of GO-based membranes has been superior to that of polymeric and nanoporous membranes because of the extraordinary characteristics of GO membranes discussed previously. In the first three subsections, we discuss the superior performance of GO membranes as reported in state-of-the-art literature for supported, self-standing, and mixed-matrix GO membranes. In the last subsection, we review the performance of GO membranes for organics and hydrocarbon separation applications.

### 4.1. Supported GO Membranes

Supported GO membranes have attracted substantial research interest because of their potential for enhancing membrane performance (i.e., permeability and selectivity) beyond the upper bounds of existing membranes [6]. Kim et al. [2] prepared graphene- or GO-supported membranes for gas separation by deposition of graphene or GO nanosheets on a poly(1-methylsilyl-1-propyne) (PTMSP) polymer. The primary pathways for gas transport in the layered graphene membrane are the defects formed during the growth of chemical vapor deposition or the wrinkles and ripples formed during the transfer of graphene film onto the polymer substrate. According to the study by Kim et al. [2], gas permeability through the graphene membrane is inversely related to the number of graphene layers deposited because of the formation of slit-like interlayer spacing, similar to that of carbon molecular sieve membranes. For example, the O_2_/N_2_ selectivity increased from 1.5 (PTMSP) to 6 (PTMSP with five interlocked graphene layers), whereas the O_2_ permeability decreased from 730 to 29 barrer (1 barrer = 10^−10^ cm^3^⋅cm/cm^2^⋅s⋅cmHg at standard temperature and pressure). In addition to preparing a PTMSP-supported membrane with stacked graphene layers, Kim et al. [2] prepared membranes with stacked GO layers (over a polyethersulfone support) with significantly larger interlayer spacing (0.6 to 1.0 nm, with the presence of a water molecule) compared with that of randomly stacked (0.355 nm) graphene layers. Graphene oxide is not completely 2-D, which results in nonuniform stacking of the GO layers and hence larger interlayer spacing. In a dry state, GO films are not permeable to small gases, such as H_2_ and He [1]. Yet because of the hydrophilic nature of GO, caused by the oxygen-containing functional groups, the interlayer spacing of GO sheets increases with the increase in humidity resulting from enhanced intercalation of the water molecules. The interlayer spacing between GO sheets can be varied by varying the degree of oxidation and the amount of intercalated water, which, in turn, allow the diffusion of selective gas molecules smaller than the 2-D nanochannels between neighboring GO sheets. 

**Figure 4:**
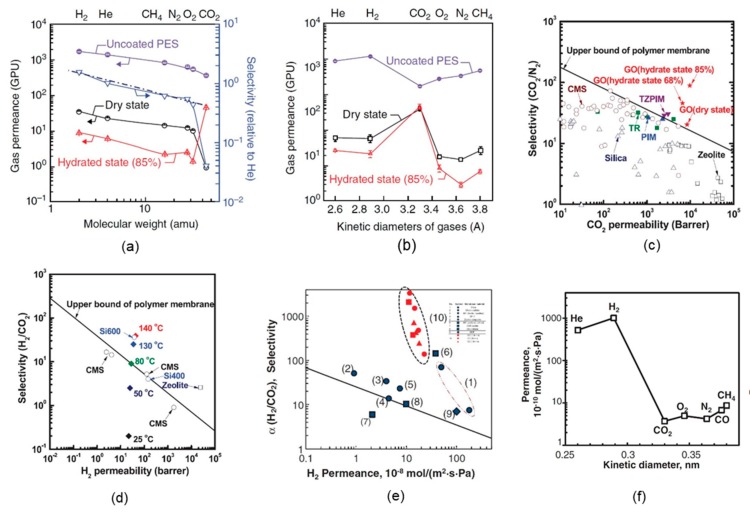
(**a**) Gas permeance values of GO membranes as a function of molecular weight (method 1) under dry and humidified conditions (dashed line represents the ideal Knudsen selectivity) [2]; (**b**) gas permeance values of GO membranes as a function of the kinetic diameter (method 2) under dry and humidified conditions [2]; (**c**) relation between CO_2_ permeability and CO_2_/N_2_ selectivity of GO membranes prepared by method 2 under dry and humidified conditions [2]; (**d**) comparison of the gas separation performance between thermally reduced GO membranes and other membranes in the literature [2]; (**e**) comparison of ultrathin GO membranes with polymeric membranes and inorganic microporous membranes for H_2_/CO_2_ mixture separation: selectivity versus H_2_ permeance (black line denotes the 2008 upper bound of the polymeric membrane for H_2_/CO_2_) [3]; (**f**) permeance values of seven molecules through an ~18 nm thick GO membrane [3]. PES, polyethersulfone support; CMS, carbon molecular sieve membrane; PIM, polymer of intrinsic microporosity; TZPIM, tetrazole PIM; TR, thermally rearranged polymer. ([a–d] From Kim, H.W.; Yoon, H.W.; Yoon, S.-M.; Yoo, B.M.; Ahn, B.K.; Cho, Y.H.; Shin, H.J.; Yang, H.; Paik, U.; Kwon, S.; Choi, J.-Y.; Park, H.B. Selective gas transport through few-layered graphene and graphene oxide membranes. *Science* 2013, *342* (6154), 91–95. Reprinted with permission from AAAS. [e, f] From Li, H.; Song, Z.; Zhang, X.; Huang, Y.; Li, S.; Mao, Y.; Ploehn, H.J.; Bao, Y.; Yu, M. Ultrathin, molecular-sieving graphene oxide membranes for selective hydrogen separation. *Science* 2013, *342* (6154), 95–98. Reprinted with permission from AAAS.).

The degree to which the GO layers interlock affects the dominant gas transport mechanism of the GO membranes. Moreover, the method used to prepare a GO membrane over a polymer support has a considerable impact on the gas permeability and selectivity of the membrane. For instance, gas transport through membranes prepared by the first method of Kim et al. [2] (described in Section 2) can be explained by Knudsen diffusion of gases in nanoporous membranes, as presented in Figure 4a. However, the selectivity of CO_2_ is significantly different from the value predicted by the Knudsen model. This result is likely due to the presence of polar carboxylic groups at the GO edges, which provide a preferential site for CO_2_ interaction. The measured permselectivity (the preferential permeation of CO_2_) of H_2_/CO_2_ for GO membranes is as high as 30, whereas the Knudsen selectivity of H_2_/CO_2_ is 4.67. When Kim et al. [2] prepared the GO membrane by direct-drop spin-casting of a GO solution, they observed a completely different set of behaviors in the gas permeance order (CO_2_ > H_2_ ≥ He > CH_4_ > O_2_ > N_2_), similar to that of high free-volume glassy polymers, as shown in Figure 4b. This method produced membranes with higher selectivity and lower permeance, exhibiting different effective diffusion pathways because of the closed-packed interlocked GO layer structure formed by the direct-drop spin-casting of the GO solution. Kim et al. [2] also observed exceptionally high CO_2_ permeance (~8500 barrer), as shown in Figure 4c, for ultrathin (<10 nm) GO membranes with a CO_2_/N_2_ selectivity of ~20 by increasing the humidity of the GO membrane. In addition, they proposed that thermal treatment of ultrathin GO membranes, which creates pores on the basal plane of the GO layer, can result in an extremely high permselectivity of 40 H_2_/CO_2_ (at ~140 °C), as shown in Figure 4d.

It is noteworthy that Karunakaran et al. [36] later reported that the extraordinary high permeability of CO_2_ (~8500 barrer) with a CO_2_/N_2_ selectivity of 20 obtained by Kim et al. [2] was based on a miscalculation of the permeance and thickness of the GO-composite membrane. When Karunakaran et al. [36] recalculated the calculations of Kim et al. using the correct permeance and thickness of the GO membrane, they found a CO_2_ permeability smaller than 1 barrer. Despite the importance of the hypothesis proposed by Kim et al. [2] and practical implementation of layered GO membranes for selective transport of gases with high permeability, further experimental verification of the performance of layered GO membranes over polymer supports is necessary to better understand the underlying nature of gas transport by GO membranes.

Ultrathin GO membranes can provide extremely high flux as well as high selectivity for the gas mixture separation [3]. Li et al. [3] reported an 18 nm thick GO membrane over an AAO support material with a 20 nm pore size that demonstrated a H_2_/CO_2_ selectivity of 3400, but with substantially low CO_2_ permeance. They showed ultrathin GO membrane whose performance was far above the upper bound of polymeric membranes, as illustrated in Figure 4e. Carbon monoxide and CH_4_ had higher permeance values than did CO_2,_ as shown in Figure 4f, even though their molecular size was larger than that of CO_2_, which is not in agreement with their proposed molecular sieving mechanism. Moreover, Li et al. [3] obtained the highest membrane performance in a 9 nm thick membrane instead of a considerably thinner (1.8 nm thin) membrane, which cannot easily be explained. One remarkable achievement of this work is that the GO membrane showed a higher separation selectivity for H_2_/CO_2_ and H_2_/N_2_, by 1 to 2 orders of magnitude, than did microporous membranes.

### 4.2. Self-Standing GO Membranes

**Figure 5:**
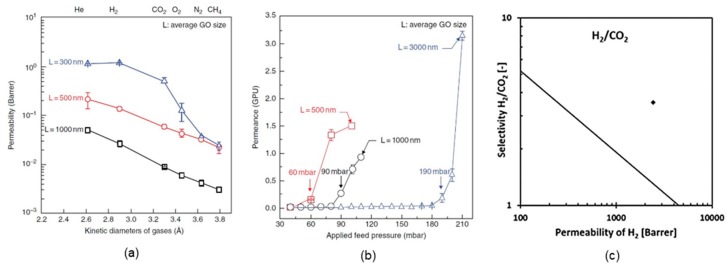
(**a**) Gas permeability of thick GO membranes with different GO platelet sizes [2]; (**b**) H_2_ permeance of thin GO-coated microporous membranes as a function of the feed pressure applied [2]; (**c**) permeability–selectivity Robeson diagrams for the gas pair H_2_/CO_2_ [4]. ([a,b] From Kim, H.W.; Yoon, H.W.; Yoon, S.-M.; Yoo, B.M.; Ahn, B.K.; Cho, Y.H.; Shin, H.J.; Yang, H.; Paik, U.; Kwon, S.; Choi, J.-Y.; Park, H.B. Selective gas transport through few-layered graphene and graphene oxide membranes [Supplementary Materials]. *Science* 2013, *342* (6154), 1–20. Reprinted with permission from AAAS. [**c**] Bouša, D.; Friess, K.; Pilnáček, K.; Vopička, O.; Lanč, M.; Fónod, K.; Pumera, M.; Sedmidubský, D.; Luxa, J.; Sofer, Z. Thin, high-flux, self-standing, graphene oxide membranes for efficient hydrogen separation from gas mixtures. *Chem. Eur. J.* 2017, *23* (47), 11416–11422. © 2017 Wiley-VCH Verlag GmbH & Co. KGaA, Weinheim. Reprinted with permission of Wiley-VCH Verlag GmbH & Co. KGaA, Weinheim).

Self-standing membranes have significant advantages over supported membranes mainly because of their overall small thickness. Kim et al. [2] experimentally obtained the effect of the average GO flake lateral size (300–1000 nm) on gas permeability in self-standing GO membranes with a thickness of ~5 µm. Park et al. [7] reproduced the results of Kim et al. [2] and systematically demonstrated (Figure 5a) that the permeability of gases increased significantly with a decrease in the average GO size. This GO size dependence was due to the shorter diffusional pathway for gases in the smaller sized GO membranes.

As illustrated in Figure 5a, the permeability of gases followed the order of the kinetic diameter of the gases (i.e., He > H_2_ > CO_2_ > O_2_ > N_2_ > CH_4_), similar to that of carbon molecular sieve membranes. The only exception observed was for the results of the 300 nm-sized GO membrane, for which the H_2_ permeability was slightly higher than the He permeability because of the higher solubility coefficient of H_2_ in porous media compared with He. Moreover, a reduction in the average GO size could significantly enhance the overall gas permeability of the membrane and reduce the required transmembrane pressure (i.e., the pressure difference between the feed and permeate side), as shown in Figure 5b. Recently, Bouša et al. [4] reported the performance of a self-standing GO membrane that was capable of high selectivity and permeability for H_2_ separation from CO_2_ and alkanes. The 15–20 µm thick GO membranes, which were defect free and mechanically stable, exhibited a H_2_/CO_2_ selectivity of 3.55 with a very high permeability of 685 barrer, as illustrated in Figure 5c [4]. This work provided additional evidence validating the concept of self-standing GO membranes. 

Gas separation through GO membranes has also been investigated through molecular simulation studies. As an example, Zheng et al. [43] recently investigated the mechanism of H_2_/CH_4_ separation through a GO membrane by using molecular dynamics simulation. While determining the diffusivity of the gas molecules through the interlayer spacing of the GO membrane, they found that the size of the gas molecule and the nature of the interaction of the gas molecule with the GO play important roles. They also observed that with the increase in interlayer spacing of the GO membrane, the H_2_ permeability first increases and then decreases, whereas the CH_4_ permeability always increases.

### 4.3. GO-Based Mixed-Matrix Membranes

**Figure 6:**
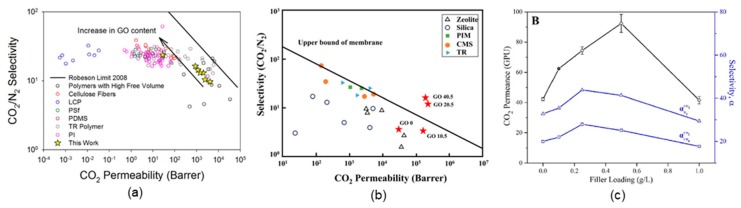
(**a**) CO_2_/N_2_ gas selectivity comparison based on a Robeson plot from 2008 [44]; (**b**) relationship between CO_2_ permeability and CO_2_/N_2_ selectivity for GO membranes in the dry state [GO 0, GO 10.5, GO 20.5, and GO 40.5 represent (PDAC/PSS)_25.5_, (PDAC/PSS)_25_(GO + GO−)_10.5_, (PDAC/PSS)_25_(GO + GO−)_20.5_, and (PDAC/PSS)_25.5_(GO/GO)_40.5_, respectively] [33]; (**c**) comparison of the gas permeance of thin film nanocomposites when using a thin film composite as the baseline [37]. LCP, liquid crystalline polymer; PSf, polysulfone; PDMS, poly(dimethylsiloxane); TR polymer, thermally rearranged polymer; PI, polyimide PIM, polymer of intrinsic microporosity; CMS, carbon molecular sieve. ([a] Reprinted from *J. Membr. Sci., 518*, Ha, H.; Park, J.; Ando, S.; Kim, C.B.; Nagai, K.; Freeman, B.D.; Ellison, C.J., Gas permeation and selectivity of poly(dimethylsiloxane)/graphene oxide composite elastomer membranes, 131–140. Copyright 2016, with permission of Elsevier. [b] Reprinted with permission of *Scientific Reports* under Creative Commons license CC BY 4.0. [c] Reprinted from *Int. J. Greenh. Gas Control*, 64, Wong, K.C.; Goh, P.S.; Ismail, A.F., Highly permeable and selective graphene oxide-enabled thin film nanocomposite for carbon dioxide separation, 257–266. Copyright 2017, with permission of Elsevier.)

To overcome the tradeoff between selectivity and permeability in polymeric membranes, nanomaterials with the desired adsorption selectivities have been added to the polymer phase (or a porous inorganic material) to obtain MMM materials, which often exhibit synergetic effects between the polymers and nanomaterials. Between the thick and thin film (selectivity layer thickness of <100 nm) membranes, thin film composites are more promising and practical for enhancing membrane performance. Previously, nanomaterials, including zeolites, carbon nanotubes, silicas, and MOF, have been considered when developing MMM materials for different applications. Because of the ultimate thinness and selective membrane performance of 2-D nanomaterial GO, it can be regarded as a promising nanofiller for different polymer membranes. To enhance the gas separation performance of PEI, Yang et al. [35] used alternately deposited GO layers with branched PEI. They employed LBL assembly, as discussed in Section 2, to alternately deposit a PEI polymer and GO nanoparticle onto a PET [poly(ethylene terephthalate)] substrate, which ensured a perfectly oriented nano-brick wall structure between the polymer and the GO. For oxygen permeability, a thin film composite of PEI and GO performed 5 or more orders of magnitude better than did traditional thick film composites. Moreover, PEI/GO films reduced the CO_2_ transmission rate by more than an order of magnitude, which provided a selectivity of H_2_/CO_2_ greater than 383. This enhanced gas barrier performance is due to the super tortuosity and diffusion length of gas molecules caused by the tightly packed nano-brick wall structure. Several facile MMM fabrication methods are available for gas separation applications. Karunakaran et al. [36] prepared GO nanocomposite membranes by embedding GO in a PEO-PBT [poly(ethylene oxide)-poly(butylene terephthalate)] copolymer for practical CO_2_ capture. They varied the GO content in the PEO-PBT polymer from 0.025 to 0.5 wt% and observed that the gas permeability values decreased with an increase in the GO content because of the high aspect ratio of the GO. The authors obtained a critical GO content (0.065 wt%) at which the CO_2_/N_2_ selectivity increased sharply (from 52 for PEO-PBT to 73 for PEO-PBT/GO) because the effect of the enhanced CO_2_ sorption capacity of the GO sheets was dominant over the reduction in gas diffusion as a result of the high aspect ratio of the GO sheets. Compared with the low GO content (0.025 to 0.5 wt%) reported in the membranes by Karunakaran et al. [36], Ha et al. [44] obtained a sharp increase in gas selectivity by using a relatively high GO content (1 to 8 wt%) in a poly(dimethylsiloxane) (PDMS)/GO composite membrane. The CO_2_/N_2_ selectivity for their membranes reached up to 24 at the maximum GO content of 8 wt% because of the enhanced selectivity of diffusion into the added GO sheets, as illustrated in Figure 6a, where the membrane performance of PDMS/GO is compared with data from several studies in the literature. Even with a very high selectivity of 24, the permeability of the membrane produced by Ha et al. [44] was considerably lower than 1000 gas permeation units (GPU). The practical requirement for postcombustion carbon capture is a CO_2_/N_2_ selectivity of 20 with a CO_2_ gas permeance of 1000 GPU. To overcome this limitation, Heo et al. [33] recently proposed a multilayer membrane development concept to prepare multilayer poly(diallyldimethylammonium chloride) (PDAC)/polystyrene sulfonate (PSS)/GO membranes that have a CO_2_/N_2_ selectivity of 15.2 and a CO_2_ gas permeance of 1175 GPU. For 20.5 and 40.5 bilayers of GO, they reported permeability values of 192,456 and 178,205 barrer, which exceed the upper bound limit of a membrane predicted by Robeson [6], as shown in Figure 6b. Moreover, the polyelectrolyte layer of PDAC/PSS improved the selectivity of CO_2_/N_2_ because the amine groups on the polymer had an affinity for CO_2_. They also observed a sharp increase in the selectivity of CO_2_ with the number of GO layers deposited on the polyelectrolyte layer. More recently, Wong et al. [37] proposed an interfacial polymerization technique for embedding thin film nanocomposite and GO layers. At a loading of 0.5 g/L of GO filler, very high separation factors for CO_2_/N_2_ (41) and CO_2_/CH_4_ (25) were achieved with a comparatively low CO_2_ permeance of 92.4 GPU, as illustrated in Figure 6c. Along with the preparation of MMM with a GO filler, some researchers have modified the GO filler properties by intercalating different MOF, such as UiO-66 [38,45], between the GO layers. The goal is to enhance the H_2_ separation performance of GO membranes by extending the gap between two GO layers, which results from the porous UiO-66-NH_2_ [38] and the reaction of the –NH_2_ group with oxygen-containing functional groups of the GO layers.

### 4.4. GO Membranes for Organics and Hydrocarbon Separation

At present, the major activity reported for the application of GO-based membranes for organic separation is organic solvent nanofiltration. Huang et al. [40] prepared a GO membrane on a ceramic hollow fiber by a vacuum suction method to separate water from organic compounds. They demonstrated excellent water separation from a dimethyl carbonate/water mixture. They passed 2.6 wt% of water in a dimethyl carbonate/water mixture through a GO membrane that was highly selective toward water. On the membrane permeate side, they obtained a water content of 95.2 wt% in the dimethyl carbonate/water mixture with a permeation flux of 1702 g m^−2^ h^−1^ through their GO membrane. Recently, Yang et al. [39] prepared very thin (~10 nm) GO laminates containing smooth 2-D capillaries made from large GO flakes for an organic solvent separation application. These membranes were suitable for organic solvent nanofiltration, which can be inferred by their >99.9% rejection of small molecular weight organic dyes dissolved in methanol. The authors suggested the nanofiltration properties of GO membranes can be enhanced because of their cation crosslinking, although this needs to be verified experimentally. 

**Figure 7:**
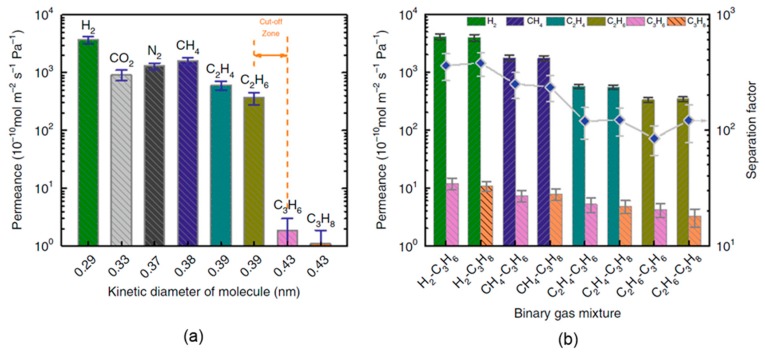
Gas permeation performance of the ED-GO@PSSHF composite membrane: (**a**) single-gas measurements of small gas molecules and light hydrocarbon permeation through the ED-GO@PSSHF membrane (Δ*P* = 2 bar, room temperature) [5]; (**b**) separation performance of the ED-GO@PSSHF membrane for binary gas mixtures (volume ratio of the mixture is 1:1, Δ*P* = 2 bar, room temperature; error bars were derived from the standard deviation) [5]. Reprinted with permission of *Nature Communications* under Creative Commons license CC BY 4.0.)

Among the very few studies on the application of GO-based membranes for hydrocarbon gas separation, Qi et al. [5] prepared a GO membrane over PSSHF by the ED method. In their GO membrane fabrication process, they performed a slight reduction of the GO, which was detrimental to the durability and quality of the membrane. Prereduction of the GO can increase disorder in the membrane by weakening its dispensability, whereas postreduction can lead to deformation of the membrane. Thus, they applied an in situ GO reduction approach by ED during membrane formation. Typically, in a dry state, GO layers have an interlayer spacing of approximately 0.43 nm. Because of this slight reduction, however, Qi et al. [5] obtained a reduced interlayer spacing of approximately 0.36 nm, which allowed molecules (H_2_, CO_2_, N_2_, CH_4_, C_2_H_4_, and C_2_H_6_) with a kinetic diameter of less than 0.39 nm to pass. The gas permeation in this case was likely dominated by Knudsen diffusion rather than molecular sieving, but it could provide a molecular sieving cutoff between the permeation of C_2_ and C_3_ hydrocarbons because of the larger kinematic diameter of propane (i.e., 0.43 nm), which is significantly larger than the GO interlayer spacing. The reported ideal selectivity values (based on single-component experiments) for C_2_/C_3_ hydrocarbons were C_2_H_4_/C_3_H_8_ = 551, C_2_H_4_/C_3_H_6_ = 319, C_2_H_6_/C_3_H_8_ = 332, and C_2_H_6_/C_3_H_6_ = 192. The gas separation performance of this strictly sieving GO membrane for propane separation is presented in Figure 7 and Table 3. The ideal selectivity between methane and propane for this ED-GO membrane was 1443.9, whereas the separation factor obtained from the binary gas mixture permeation was 234.7.

Table 4 compares the performance of all the gas separation membranes discussed in this review and shows that the GO-based membrane technology has the potential to overcome the limitation of sacrificing permeability to obtain better selectivity. Both nanocomposite GO membranes and supported GO membranes are promising options for achieving higher selectivity as well as higher permeability for the separation of relatively small gas molecules from a gas mixture.

Reprinted with permission of *Nature Communications* under Creative Commons license CC BY 4.0.

## 5. Conclusions

Graphene oxide membranes can provide extremely high fluxes (i.e., at least 1 order of magnitude higher than the conventional materials) mainly because of their ultimate thinness. In addition, they can have high selectivity because of the molecular sieving or diffusion effect resulting from their narrow pore size distribution or their unique surface chemistry. Gas molecules can travel either through the defects in a GO layer generated during GO preparation or through the interlayer spacing between GO sheets. One of the most important characteristics of the layered GO membrane is the interlayer spacing between GO sheets, which can be tailored for optimal transport of the desired molecules through the membrane. Graphene oxide membranes can be prepared and engineered in several ways, as summarized and briefly analyzed below:

- Graphene oxide-supported membranes can be prepared by a facile method through the deposition of GO on various support materials, such as a porous polymer, porous metal, or porous ceramic. They can provide enhanced gas separation performance while having reasonable mechanical stability. Proper selection of the membrane support materials, with consideration given to their porosity, thickness, thermal stability, wettability, and compatibility with the application environment, is critically important. Polyethersulfone is among the best flexible porous polymeric support materials identified in this review. Porous stainless-steel substrates and porous ceramic membranes could also be used as suitable support materials for the fabrication of GO-supported membranes. However, the relatively large thicknesses of these nonpolymeric support materials might pose a limit to the flux of gas transport through the membrane.
- The self-standing GO membrane concept was developed based on the target of achieving an ultrathin membrane. However, the main issue that needs to be resolved is the mechanical stability and robustness of self-standing membranes.
- Nanocomposite GO membranes, prepared by a composite of GO nanosheets and a suitable polymeric or inorganic membrane material, is one emerging technology used to develop membranes with higher permeability and higher selectivity beyond the upper bounds of existing polymeric and nanoporous membranes. The preparation of ultrathin nanocomposite membranes might be challenging, considering the limits for preparing thin inorganic membranes with acceptable mechanical properties.
- A GO membrane in a dry state has an interlayer spacing of 0.43 nm, which can be further reduced by the controlled reduction and removal of oxygen functionalities or can be increased by intercalation with water or other molecules. This approach would enable the GO membrane to be tailored for specific gas separation applications.

On the basis of data reported on the performance of GO-based membranes for gas separation applications, the GO-based membrane technology has the potential to overcome the limitation of sacrificing permeability to obtain better selectivity. Controlling the interlayer spacing of GO layers is vital when developing a gas separation membrane to selectively separate a target gas from a gas mixture. Several approaches can be adopted to control the interlayer spacing of the GO layers in the GO-PES membrane, such as thermal reduction of graphite oxide powder prior to the exfoliation to monolayer GO, in situ chemical reduction of the GO solution by vacuum filtration while preparing the membrane, and postreduction of the GO-PES membrane with a chemical reagent. Selecting the proper support materials (for GO-supported membranes) or developing suitable composite materials (for GO-nanocomposite membranes) as well as innovative fabrication methods can lead to the development of high-performing, robust GO membranes that can tolerate challenging process conditions (i.e., high temperature and pressure, corrosive conditions, and various humidity levels) while providing high selectivity and high flux, beyond the achievable limits of the existing membrane materials.

## Figures and Tables

**Table 1:** Graphene oxide (GO)/support material fabrication methods (gas separation applications) ^a^.

GO/Support Material	Preparation Method	Reference
GO/PES	Dip casting, drop coating	[2]
GO/AAO	Vacuum filtration	[3]
GO/Cu	Spin coating, spray coating, vacuum filtration	[1]
Highly laminated GO	Ultrasonic exfoliation, stepwise separation	[39]
Self-standing GO	Gravitation assembly	[4]
GO/ceramic hollow fiber	Vacuum suction	[40]

^a^ PES, polyethersulfone; AAO, anodic aluminum oxide.

**Table 2:** Graphene oxide (GO)/support material characterization techniques (gas separation applications) ^a^.

Characterization Approach	Measurements	References	Major Findings
SEM (FE-SEM)	Size and distribution of the GO flakes	[2,3,4,5,41]	GO particle size of 0.1–20 µm
TEM	Thinness and curliness of the GO nanosheets	[2,5,41]	Effectiveness of the method of exfoliation
AFM	Surface morphology and depth profile of the GO membrane	[2,3,4,5,34,41]	Thickness of a single GO layer of 0.7–0.9 nm
XPS	Surface chemical composition	[3,4,5]	Density of different bonds, e.g., C = C (28.04%), C–C (13.79%), C = O (27.11%), C–O (14.62%), O–C = O (12.52%)
XRD	Amount of GO content and curvature in the polymeric support for GO membranes	[4,5,41]	GO has a peak 2θ at 11.1°
FTIR	Analysis of the functional groups	[4,5,41]	Functional groups in the form of stretching vibrations of different bonds, e.g., O–H (3400 cm^−1^), C = O (1720 cm^−1^), C = C skeletal (1620 cm^−1^), C–O carboxyl (1390 cm^−1^), C–O epoxy (1195 cm^−1^), and C–O alkoxy (1035 cm^−1^)
Raman spectroscopy	Structural integrity of the GO membrane	[3,4,5,41]	GO powder: G band at 1585 cm^−1^ and D band at 1338 cm^−1^
WCA	Wettability of the GO membrane	[5]	GO membrane WCA of 46.7 ± 1.1°
DLS	Particle size distribution	[34]	Particle size distribution of the GO membrane, e.g., 10 µm (30%), 20 µm (40%)
TGA	Weight percentage of GO or a GO–polymer composite with variation in temperature	[41]	Weight percentage of GO or a GO–polymer composite at different temperatures

^a^ SEM, scanning electron microscopy; FE-SEM, field emission scanning electron microscopy; TEM, transmission electron microscopy; AFM, atomic force microscopy; XPS, X-ray photoelectron spectroscopy; XRD, X-ray diffraction; FTIR, Fourier-transform infrared spectroscopy; WCA, water contact angle; DLS, dynamic light scattering; TGA, thermogravimetric analysis.

**Table 3:** The ideal selectivity and separation factors of small gas molecules and light hydrocarbons over the ED-GO@PSSHF membrane (Δ*P* = 2 bar, room temperature) [5].

Ideal Selectivity (row/col.) ^a^	H_2_ (0.29 nm)	CO_2_ (0.33 nm)	N_2_ (0.37 nm)	CH_4_ (0.38 nm)	C_2_H_4_ (0.39 nm)	C_2_H_6_ (0.39 nm)	C_3_H_6_ (0.43 nm)
CO_2_ (0.33 nm)	4.1						
N_2_ (0.37 nm)	2.9	0.7					
CH_4_ (0.38 nm)	2.3	0.6	0.8				
C_2_H_4_ (0.39 nm)	6.1	1.5	2.1	2.6			
C_2_H_6_ (0.39 nm)	10.1	2.5	3.6	4.3	1.7		
C_3_H_6_ (0.43 nm)	1949.2 (361.5) ^b^	478.9	683.8	836.0 (249.7) ^b^	319.1 (119.6) ^b^	192.3 (84.6) ^b^	
C_3_H_8_ (0.43 nm)	3366.8 (378.7) ^b^	827.2	1181.1	1443.9 (234.7) ^b^	551.1 (121.8) ^b^	332.2 (121.2) ^b^	1.7

^a^ The kinetic diameter of the responding molecules. ^b^ The separation factors obtained from the binary gas mixture permeation.

**Table 4:** Summary of graphene oxide (GO) membrane performance for gas separation applications ^a^.

Type of GO Membranes	GO/Supporting Material	Application	Membrane Performance	Reference
Polymer-supported GO membranes	GO/PES	CO_2_ separation	a. CO_2_/N_2_ selectivity: 20	[2]
			b. CO_2_ permeability: 8500 barrer (1 barrer = 1 × 10^−10^ cm^3^⋅cm/cm^2^·sec·cmHg)	
	GO/AAO	H_2_ separation	a. H_2_/CO_2_ selectivity: 3400	[3]
			b. H_2_/N_2_ selectivity: 900	
Self-standing GO membranes	GO (~5 µm)	H_2_ separation	CO_2_ permeability: <0.5 barrer	[2]
	GO (15–20 µm)	H_2_ separation	a. H_2_/CO_2_ selectivity: 3400	[4]
			b. CO_2_ permeability: 685 barrer	
GO-based mixed-matrix membranes	PEI/GO	CO_2_ separation	a. H_2_/CO_2_ selectivity: >383	[35]
			b. O_2_ permeability: 2.5 × 10^−20^ cm^3^⋅cm⋅cm^−2^⋅s^−1^ Pa^−1^	
	PEO-PBT/GO	CO_2_ separation	a. CO_2_/N_2_ selectivity: 73	[36]
			b. CO_2_ permeability: 143 barrer	
	PDMS/GO	CO_2_ separation	a. CO_2_/N_2_ selectivity: 24	[44]
			b. CO_2_ permeability: <1000 GPU	
	(PDAC/PSS)/(GO/GO)	CO_2_ separation	a. CO_2_/N_2_ selectivity: 73	[33]
			b. CO_2_ permeability: 143 barrer	
	UiO-66-NH_2_/GO	H_2_ separation	a. H_2_/N_2_ selectivity: 9.75	[38]
			b. CO_2_ permeability: 6.1 × 10^−9^ mol⋅m^−2^⋅s^−1^⋅Pa^−1^	
	TFN/GO	CO_2_ separation	a. CO_2_/N_2_ selectivity: 41	[34]
			b. CO_2_ permeability: 92.4 GPU	
Hydrocarbon mixture separation	GO/PSSHF	Hydrocarbon separation	a. Ideal selectivity between methane and propane 1443.9	[3]
			b. Separation factor of methane/propane from binary gas mixture 234.7	

^a^ PES, polyethersulfone; AAO, anodic aluminum oxide; PEI, polyethylenimine; PEO, poly(ethylene oxide); PBT, poly(butylene terephthalate); PDMS, poly(dimethylsiloxane); PDAC, poly(diallyldimethylammonium chloride); PSS, polystyrene sulfonate; UiO-66-NH_2_, functionalized zirconium-based porous metal–organic frameworks; TFN, thin film nanocomposite; MOF-5, zinc-based metal–organic frameworks; PSSHF, porous stainless steel hollow fibers.

## Abbreviations

AAO	anodic aluminum oxide
AFM	atomic force microscopy
CMS	carbon molecular sieve
DLS	dynamic light scattering
ED	electrophoresis deposition
FE-SEM	field emission scanning electron microscopy
FTIR	Fourier-transform infrared spectroscopy
GO	graphene oxide
LB	Langmuir–Blodgett
LBL	layer by layer
MMM	mixed-matrix membranes
MOF	metal−organic frameworks
MOF-5	zinc-based metal−organic frameworks
PBT	poly(butylene terephthalate)
PDAC	poly(diallyldimethylammonium chloride)
PDMS	poly(dimethylsiloxane)
PEI	polyethylenimine
PEO	poly(ethylene oxide)
PES	polyethersulfone
PIM	polymer of intrinsic microporosity
PSS	polystyrene sulfonate
PSSHF	porous stainless steel hollow fibers
SEM	scanning electron microscopy
TEM	transmission electron microscopy
TFN	thin film nanocomposite
TR polymer	thermally rearranged polymer
TGA	thermogravimetric analysis
TZPIM	tetrazole polymer of intrinsic microporosity
UiO-66-NH_2_	functionalized zirconium-based porous metal–organic frameworks
WCA	water contact angle
XPS	X-ray photoelectron spectroscopy
XRD	X-ray diffraction
